# The Use of Interferon Gamma Release Assays in the Diagnosis of Active Tuberculosis

**DOI:** 10.1155/2012/768723

**Published:** 2012-01-22

**Authors:** Silvan M. Vesenbeckh, Nicolas Schönfeld, Harald Mauch, Thorsten Bergmann, Sonja Wagner, Torsten T. Bauer, Holger Rüssmann

**Affiliations:** ^1^Department of Pneumology, Lungenklinik Heckeshorn, HELIOS Klinikum Emil von Behring, 14165 Berlin, Germany; ^2^Institute of Microbiology, Immunology and Laboratory Medicine, HELIOS Klinikum Emil von Behring, 14165 Berlin, Germany

## Abstract

Interferon gamma release assays (IGRAs) are *in vitro* immunologic diagnostic tests used to identify *Mycobacterium tuberculosis* infection. They cannot differentiate between latent and active infections. The cutoff suggested by the manufacturer is 0.35 IU/mL for latent tuberculosis. As IGRA tests were recently approved for the differential diagnosis of active tuberculosis, we assessed the diagnostic accuracy of the latest generation IGRA for detection of active tuberculosis in a low-incidence area in Germany. Our consecutive case series includes 61 HIV negative, *Mycobacterium tuberculosis* culture positive patients, as well as 234 control patients. The retrospective analysis was performed over a period of two years. In 11/61 patients with active tuberculosis (18.0%) the test result was <0.35 IU/mL, resulting in a sensitivity of 0.82. We recommend establishing a new cut-off value for the differential diagnosis of active tuberculosis assessed by prospective clinical studies and in various regions with high and low prevalence of tuberculosis.

## 1. Introduction

Tuberculosis (TB) remains a major public health problem affecting one-third of the world's population [1, 2]. Diagnosis of TB is usually based on a combination of anamnestic symptoms, clinical presentation, radiological and pathological changes, bacteriological findings of acid/alcohol-fast bacilli, and molecular tests [3]. Definitive TB diagnosis is based on the detection of *Mycobacterium tuberculosis *(MTB) in the culture, which usually takes four to six weeks. For decades, tuberculin skin test (TST) has been used as diagnostic tool to support the physician's decision process. With the introduction of interferon gamma release assays (IGRAs), a more specific method became available. Although primarily developed for the diagnosis of latent TB, clinicians have also been searching for improved diagnostic tools and explored IGRAs for the immunodiagnosis of active TB. In 2010, the Centers for Disease Control and Prevention (CDC) updated their guidelines for testing for TB infection, concluding that IGRAs “may be used instead of a tuberculin skin test in all situations in which the CDC recommends the tuberculin skin test as an aid in diagnosing *M. tuberculosis* infection” [4, 5].

Nevertheless, with the cutoff for the diagnosis of latent TB as given by the producers, pooled sensitivity for the diagnosis of culture positive TB did not exceed 80% in the most recent meta-analyses [6, 7]. The present case series constitutes one of the largest reports of latest generation IGRA used in culturally proven HIV-negative TB cases in a low-prevalence country. Our study is meant to help evaluate the cutoff for the IGRA in the differential diagnosis of TB.

## 2. Study Population and Methods

This is a retrospective study performed on inpatients of a regional hospital specialized in lung diseases (Lungenklinik Heckeshorn, Berlin). At least one IGRA is routinely performed on a blood sample of each TB-suspect patient, and every suspicious sample (smear, lymph node biopsy, pleural effusion, or biopsy) is routinely cultured for TB. All samples of patients included in this study were taken prior to initiation of antibiotic therapy. Over a two-year period (1/2008–1/2010), IGRA results of all MTB culture positive cases of active TB were analyzed. Patients with other lung diseases than TB, including negative history for MTB infection and without radiological findings suggestive of MTB infection in the past and with no signs of active disease, were chosen as control group. All HIV-positive patients were excluded from the database.

### 2.1. IGRA

We used the latest generation IGRA (QuantiFERON-TB Gold in-Tube, Cellestis, Carnegie, Australia), later referred to as QFT-GIT, on all samples. Peripheral blood samples were obtained by trained personnel in specific blood collection tubes following the manufacturer's instructions. All blood samples were processed within 4 h of phlebotomy. Otherwise, the test was performed as previously described [8].

### 2.2. TB Culture

Specimens were stained, processed, and cultured by standard procedures in mycobacteriology [9]. The isolates were cultured for 4 weeks on Löwenstein-Jensen (L-J) medium at 37°C and tested for growth rate, pigment production, and by biochemical testing using standard methods [10, 11].

### 2.3. Statistical Analysis

Data were analyzed using STATA 12.0 (StataCorp, College Station, Texas, USA). The *t*-test for independent samples (two-tailed) was carried out to assess significance level of detected differences in both groups (IGRA test results, age).

## 3. Results

**Figure 1:**
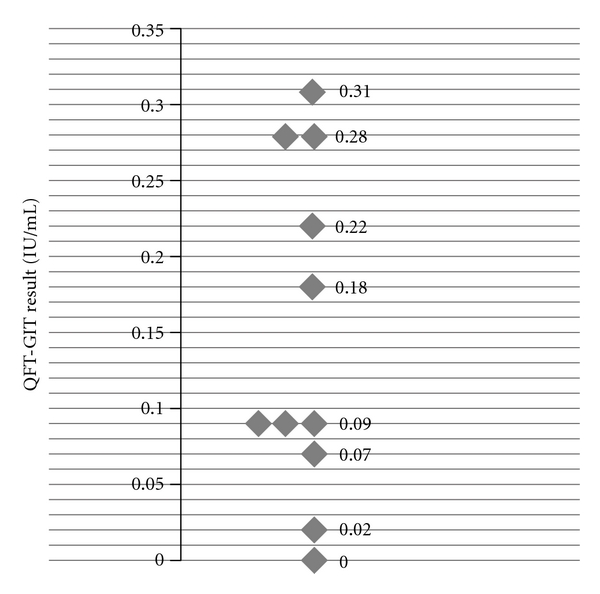
Range of QFT-GIT values below the cutoff of 0.35 IU/mL for patients with active tuberculosis.

A total of 61 patients with active TB as ascertained through positive MTB culture were examined using QFT-GIT between 1/2008 and 1/2010 (see Table 1(a)). 53 patients presented with pulmonary TB, three patients with lymph node TB, and five patients with tuberculous pleurisy. The median QFT-GIT value for all TB patients was 3.46 IU/mL, ranging from 0.00 to 300 IU/mL. In 11 patients, the test result was <0.35 IU/mL (see Figure 1). Assuming the cutoff for latent TB suggested by the producer, these are false-negative (FN) test results (11/61 = 0.18; 18% FN). We calculated the sensitivity (the proportion of patients with active TB who are correctly identified as such) as 1-FN (=82%). Only in one patient four months after therapy with adalimumab, the result was 0.00 IU/mL, and in one other patient with lung cancer under concomitant chemotherapy, it was 0.02 IU/mL. All other patients showed values of 0.07 IU/mL or above. The median age of our patients was 46 years (range: 5–84 years), in the group with a test result ≥0.07 and <0.35 IU/mL, the median age was 80 years (range: 44–84 years), and, in the group testing ≥0.35 IU/mL, it was 36 years (range: 5–84 years).

Within the control group (see Table 1(b)), QFT-GIT values ranged from 0.00 to 103.4 IU/mL in one patient with pneumonia. The median was 0.00 IU/mL. In 50% of all cases (116/234), no Interferon gamma (IFN-*γ*) release was measured (0.00 IU/mL). In 51/234 patients (21%), test results were above 0.35 IU/mL. The median age in the control group was 66 years with a range from 1 to 99 years.

Patients in the study group were significantly younger than in the control group and had significantly higher QFT-GIT test results (*P* < 0.05 for both, see Table 1(c)).

## 4. Discussion

IGRAs are *in vitro* immunologic diagnostic tests to identify MTB infection. Latent and active infections are not differentiated. Several test systems are commercially available: QuantiFERON-TB Gold (QFT), QuantiFERON-TB Gold in-Tube (QFT-GIT), both Cellestis Limited, Chadstone, Australia, and T-SPOT.TB (Oxford Immunotec, Abingdon, UK). The QFT-GIT measures IFN-*γ* responses to the MTB specific antigens early secretory antigenic target 6 (ESAT-6), culture filtrate protein 10 (CFP-10), and Rv2654 (TB 7.7). Their use in clinical practice is more and more widespread. Whereas the test was initially conceived to support diagnosis of latent infection, an increasing body of evidence is published on its use in detection of active TB infection [12–14]. More and more guidelines now include recommendations for or against the use of IGRAs in the differential diagnosis of active TB [15].

Several systematic reviews and meta-analyses have recently been published specifically on the diagnostic accuracy of IGRAs in active TB [6, 16, 17]. Strong heterogeneity between study populations is a major limitation of these meta-analyses, and most studies had small sample sizes. Overall, few studies were done with the latest generation QFT-GIT in areas of low endemicity such as Germany (2 studies in [17], 13 studies in [16]). Among those, even fewer restricted sensitivity analysis to patients with culturally proven MTB infection and confirmed HIV-negative status [12, 18].

Specificity depends highly on the definition of the control group. Pai et al. reported a pooled specificity of 99% among non-BCG vaccinated and 96% among BCG-vaccinated low-risk groups [17]. According to a recent meta-analysis that did not restrict studies on specificity to low-risk groups [6], a situation more compatible to our clinical setting, the specificity of QFT-GIT was only 0.79 (95% CI 0.75–0.82). In our study, 51 out of 234 control patients (21%) showed test results above 0.35 IU/mL, indicative of latent TB according to the producer. However, this finding is also consistent with the expected number of false positives assuming a specificity of around 0.8 according to Sester et al. [6]. The retrospective design of our study does not allow any conclusion about the test specificity in our study population.

Sensitivity in these studies was also found to be highly dependent on the study population, notably local TB prevalence, and ranged from 0.58 in a high-prevalence country [19] to 1.00 in a low-prevalence country [20], when QFT-GIT was assessed. Diel et al. found a pooled sensitivity of 0.84 (95% CI 0.81–0.87) when including only developed countries [16], consistent with the results published by Sester et al. (0.77, 95% CI 0.75–0.80) [6].

In our consecutive case series of 61 TB culture positive, HIV-negative patients in a low-prevalence setting in Germany, we found a sensitivity of 82.0% (95% CI: 0.696, 0.902) for the QFT-GIT, when the cutoff recommended for the diagnosis of latent TB was used (<0.35 IU/mL). In a low-prevalence country such as Germany with a TB prevalence of 5.4/100,000 inhabitants [21], case finding is an outstanding priority in the management of this disease. Therefore, highly sensitive test systems are needed for screening purposes. If the use of IGRA in the differential diagnosis of active TB is recommended, at least under certain conditions, then the cut-off point for active TB should be lower compared to latent TB, as previously discussed by Davidow [22]. Prospective clinical studies in different defined regions, with high- and low-prevalence of active TB, should be performed to evaluate the use of IGRAs in the diagnostic workup of TB patients. The cutoff for the detection of latent TB does not seem applicable for that purpose.

## 5. Conclusions

We suggest that the cutoff for the use of IGRA in the differential diagnosis of active TB in low incidence settings be reevaluated. Further prospective studies including clinical criteria for TB are needed to determine a new cutoff for active TB.

## Figures and Tables

**Table 1a:** (a)

MTB infection	Male	Female	Total	Median age, years (range)	Median QFT, IU/mL (range)
Lung	28	25	53	45 (4–83)	2.40 (0,00–103,4)
Pleura	2	3	5	26 (17–76)	2,45 (0,96–300)
Lymph node	2	1	3	65 (25–69)	21,32 (3,78–86,8)

Total	32	29	61	46 (5–84)	3.46 (0,00–300)

**Table 1b:** (b)

Diagnosis	Male	Female	Total	Median age, years (range)	Median QFT, IU/mL (range)
Malignancy	22	23	45	69 (39–90)	0,01 (0,00–23,9)
Bronchitis	27	13	40	64 (1–90)	0,00 (0,00–1,84)
Pneumonia	30	17	47	67 (1–99)	0,00 (0,00–75)
Others	53	49	102	62,5 (5–99)	0,02 (0,00–103,4)

Total	132	102	234	66 (1–99)	0,00 (0,00–103,4)

**Table 1c:** (c)

	*n*	QFT			Age		
		Mean	95% CI	*P*	Mean	95% CI	*P*
TB	61	14.13	3.44–24.82	<0.05	47.8	41.88–53.79	<0.05
Control	234	1.33	0.24–2.43		63	60.56–65.39	
